# Improving the Timeliness and Safety of Therapeutic Hypothermia for Hypoxic–Ischemic Encephalopathy

**DOI:** 10.1097/pq9.0000000000000283

**Published:** 2020-05-12

**Authors:** Hannah N. Smith, Colleen A. Hughes Driscoll

**Affiliations:** From the * University of Maryland School of Medicine, Baltimore, Md.; † Department of Pediatrics, University of Maryland School of Medicine, Baltimore, Md.

## Abstract

**Introduction:**

Therapeutic hypothermia (TH) is a time-sensitive, efficacious treatment for newborns who experience perinatal hypoxic–ischemic encephalopathy. Optimal management of patient temperatures during TH may improve newborn outcomes and reduce side effects. We noted that patients undergoing TH were often outside of the target temperature range during treatment. This project sought to improve the timely initiation of effective treatment and temperature stability during TH through system-based changes in practice.

**Methods:**

Measures include the time to target temperature, the percentage of core temperatures outside of the target range, and the absolute difference between core and peripheral temperatures over 41 months. System-based changes in the TH protocol included changing from passive to active hypothermia on transport and utilizing a delivery mode that uses more gradual temperature fluctuations during TH. We compared measures of health status and side effects as balancing measures.

**Results:**

The TH protocol changes resulted in a significant reduction of time to goal temperature from 1.67 to 0.49 hours, in the percentage of temperature readings outside goal range from 12.6% to 6.3%, and the average absolute difference between core and peripheral temperatures from 1.78°C to 1.47°C. No adverse health outcomes were detected. We observed decreases in vasopressor use with each protocol change.

**Conclusions:**

This study demonstrates that detailed attention to the method of delivery of TH has an impact on ensuring effective delivery of therapy and minimizing the risks of treatment. The protocol changes were not associated with an increase in adverse events and were associated with a reduction in vasopressor use.

## INTRODUCTION

Therapeutic hypothermia (TH) is a proven treatment to reduce long-term morbidity and remains a standard of care in appropriately selected patients with perinatal hypoxic–ischemic encephalopathy (HIE).^1–4^ The timing and safe delivery of TH is essential for the impact it has on reducing the rates of death or survival with a disability; evidence suggests that earlier treatment is associated with more positive outcomes.^5–10^

Although TH is both a safe and efficacious treatment for perinatal HIE, there are associated side effects of treatment. Bradycardia, hypotension, subcutaneous fat necrosis, and coagulopathies are among the associated complications.^11^ Patients receiving TH should be kept within the clinically tested therapeutic range of 33°C−34°C to optimize the benefits of neuroprotection while limiting the risk of side effects and avoiding significant differences between core and peripheral temperatures.^11,12^ We observed that patients undergoing TH at our institution were often outside of the target temperature range during treatment, both on admission to our neonatal intensive care unit (NICU) and during the TH treatment.

This single-center quality improvement (QI) project aimed to optimize the benefits and reduce the harms of TH by decreasing the time for patients to achieve target core temperatures, decreasing core temperatures outside the therapeutic window during TH, and decreasing the difference between core and peripheral temperatures. This study tested 2 systematic practice changes aimed at improving temperature regulation for HIE patients in our NICU. The first practice change was a change from passive to active TH during the transport of newborns born at outlying institutions. The second practice change was to promote more gradual temperature changes by modification of the servo-control mode on the cooling device, Blanketrol III (Cincinnati Sub-Zero Products, Cincinnati, Ohio). We hypothesized that these interventions would lead to shorter times to goal temperature, smaller differences in the core to peripheral temperatures, and a decrease in the number of temperature readings outside of the target range. We aimed to reduce the time to target temperature and the percentage of temperatures that fell outside of the target range by 50% within 6 months.

## METHODS

The University of Maryland Baltimore Institutional Review Board determined that this quality improvement project was not Human Subjects Research. Therefore, the Board’s review and approval were not required.

### Setting and Patient Population

We performed this project in a level IV, 52-bed NICU within an urban, academic medical center. The institution has a high-risk birthing center that delivers approximately 1,700 newborns annually. The hospital is 1 of 2 statewide referral centers for providing TH to patients with HIE. Both referral centers operate a combined neonatal transport team with ground and air services to provide TH for patients from outlying hospitals during transport.

### Preintervention Era

From January 2016 to August 2017, patients transferred for TH from outlying hospitals were passively cooled by discontinuation of radiant heat and unbundling, while monitoring peripheral temperatures. If a patient’s temperature fell below a threshold, the transport team would implement maneuvers to warm the patient actively. Upon admission to the NICU, patients received TH with the Blanketrol III cooling blanket, a servo-controlled water-based blanket for whole-body regulation of temperature. Core temperatures were monitored with an esophageal probe, whereas peripheral temperatures were monitored with a skin probe, using the Auto mode setting.

### Protocol Changes

Our first protocol change occurred in August 2017, by replacing passive TH with active TH for transported patients using the Tecotherm cooling device (Inspiration Healthcare Group, Crawley, United Kingdom). This change was made based on evidence from other NICUs demonstrating that active cooling on transport with a servo-controlled device decreased the time to goal therapeutic core temperature range and temperature stabilization.^12,13^

In August 2018, a second protocol change was made based on the observation that some patients who received TH spent a notable period with peripheral temperatures outside of the target range, regardless of having been transported or being inborn. To further understand the potential improvements that could be made to our TH protocol, we reviewed all aspects of our practice, including the mode of the TH device, Blanketrol III. Based on evidence indicating that the device mode can impact how quickly patients can achieve and maintain temperatures within the target range,^14,15^ we changed the Blanketrol III device settings from Auto mode to a combination of Gradient 10 and SMART mode. The Auto mode will increase or decrease the temperature of the water in the blanket to extremes to adjust the patient’s core temperature to the desired set point rapidly. Gradient 10 and SMART mode theoretically allow for a more gradual adjustment by increasing or decreasing the blanket temperature by 5° to 10° increments from the patient’s core temperature until the desired core temperature is achieved.^16^ Patient temperatures during TH were reviewed on a case basis monthly during a divisional Quality Assurance/Quality Improvement conference to monitor outcomes, highlight protocol changes, and address issues with protocol implementation.

### Population, Eras, and Measurements

This study included early-term and full-term newborns who met the institutional TH inclusion criteria and were treated for HIE between January 2016 and May 2019. We excluded patients if active cooling was initiated at an outside hospital or if they had chromosomal or severe congenital abnormalities as these could interfere with typical thermoregulation strategies. Also, patients were excluded if the institutional standard protocol was not followed, if there was inadequate documentation of core temperatures in the electronic medical record, or if they did not achieve stable temperatures before transfer out of the NICU. The study covers 3 eras including era 1 or the preintervention period (January 2016 to July 2017), era 2 marked by the institution of active cooling on transport (August 2017 to July 2018), and era 3 marked by the institution of Gradient 10 and SMART modes (August 2018 to May 2019).

We identified 3 primary outcome measures. Time to achieve the target temperature was defined as the time between the start of active in-hospital TH to the first core temperature within the target range. The second primary outcome was the average absolute difference between peripheral and core body temperatures during TH. As the peripheral temperature during TH can be higher or lower than the core temperature, the absolute difference was used for calculations. A reduction in the absolute difference would indicate more controlled temperature management. The third primary outcome was the percentage of core temperatures that were outside of the target range during TH. Temperatures that fell both above and below the target temperature range were relevant as temperatures below the target range can increase the risk of side effects of therapy, whereas temperatures above the range can reduce the efficacy of therapy. We compared laboratory tests related to coagulation times, liver and kidney function, and blood cell abnormalities as balance measures to ensure that the TH protocol changes did not result in any unintended health outcomes for patients undergoing treatment. Potential side effects of TH, including subcutaneous fat necrosis and bradycardia noted during the hospitalization, were compared across eras. Health factors that may represent the severity of illness were also assessed for each patient group, both to determine equipoise and to evaluate for unintended consequences of our practice change.

### Data Collection and Analysis

Data collected included demographics, monitored temperature readings, and laboratory values relevant to side effects and symptoms of HIE and TH. Temperatures were recorded in the electronic medical record, as part of our institutional TH protocol. The time to target temperature and the absolute difference between core and peripheral temperatures for each patient were analyzed on a run chart, using accepted run chart interpretation rules.^17^ For the remaining analyses, we analyzed continuous variables using *t* tests; discrete data were analyzed using the chi-square test. Fischer–Freeman–Halton Exact tests were utilized for discrete data when the sample sizes and distributions were not appropriate for the use of chi-square tests. Analysis of variance was used to compare baseline demographic data and laboratory results. We analyzed data using Microsoft Excel (Microsoft Corp., Redman, Wash.) and SPSS (IBM Corp,., Armonk, N.Y.).

## RESULTS

### Demographics

A total of 69 patients received TH during the study period. We excluded 15 patients: 2 patients for having a chromosomal abnormality, 2 patients for being transferred out of the NICU without stabilization of temperatures, 2 for active cooling initiated at an outside hospital, and 9 for inadequate documentation. Of the qualifying infants, 26 patients received TH in era 1, 13 in era 2, and 15 in era 3. Patient demographics are shown in Table 1. Gestational ages and birth weights of the patients were similar across all 3 time periods. Patients’ Sarnat scores,^18^ Apgar scores, and worst blood gas pH were not statistically different when compared across eras, and there were no differences in the percentage of patients who were transported versus inborn.

**Table 1.**
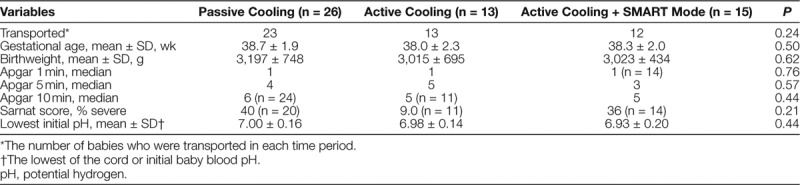
Admission Demographics and Measures of Initial Condition Severity by Protocol

### Primary Outcomes and Balancing Measures

**Fig. 1.**
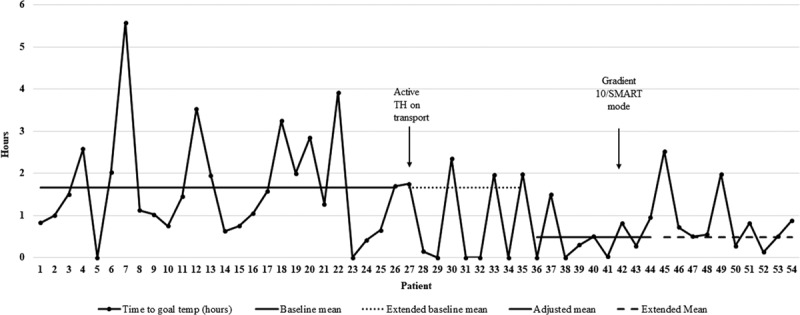
Annotated run chart showing time to target temperature. Arrows indicate TH protocol changes.

**Fig. 2.**
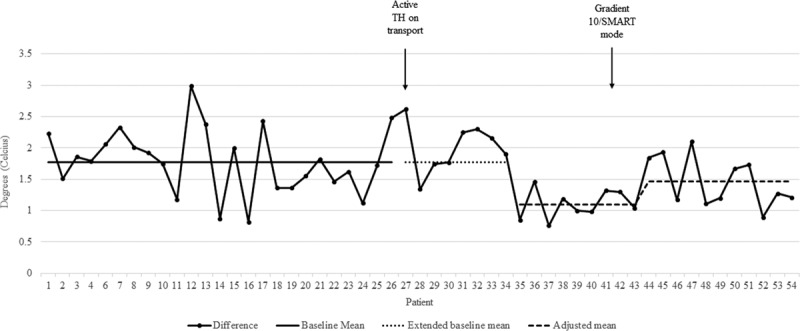
Annotated run chart showing the mean difference between core and peripheral temperatures. Arrows indicate protocol changes.

The time to achieve target temperatures significantly decreased 9 months following the change to active cooling with a mean from 1.67 to 0.49 hours (Fig. 1). This change was maintained over the remainder of the study period. The mean absolute difference between core and peripheral temperatures significantly decreased 8 months following our first intervention from 1.78°C to 1.10°C. Following the change to Gradient 10/SMART modes, there was a small, but significant, increase in the temperature difference to 1.47°C (Fig. 2). Because we observed a shift in an unexpected direction (starting at patient 43) that coincided roughly with the change in the mode of the Blanketrol, we were unclear if the special cause variation was attributable to the change in the mode or to another unidentified cause. To address this, we performed a post hoc analysis to compare mean absolute temperature differences between patients exposed to Auto mode (era 2, patients 27−41) versus patients exposed to Gradient 10/SMART mode (era 3, patients 42−54). In this analysis, the mean absolute difference between core and peripheral temperatures decreased significantly in those exposed to Gradient 10/SMART mode (1.4°C versus 1.6°C, *P* = 0.001).

The percentage of core temperature readings during treatment that was outside of the target range significantly decreased from 12.6% to 10.5% between eras 1 and 2 and significantly decreased again to 6.3% in era 3 (Table 2).

**Table 2.**
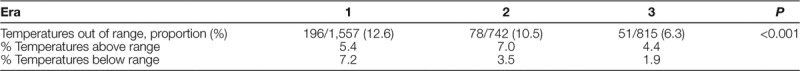
Temperature Readings Outside Therapeutic Range

Measurements of coagulation times, liver and kidney function, and blood cell abnormalities were consistent across eras. Additionally, the percentage of patients who had g-tubes, tracheostomies, seizures, arrhythmias, bradycardia, or fat necrosis did not differ across periods (Tables 3 and 4). The proportion of deaths was also similar across eras. The percentage of infants who received vasopressor support significantly decreased from 46.2% to 30.8% from era 1 to era 2 and decreased further to 6.7% in era 3 (Table 4).

**Table 3.**
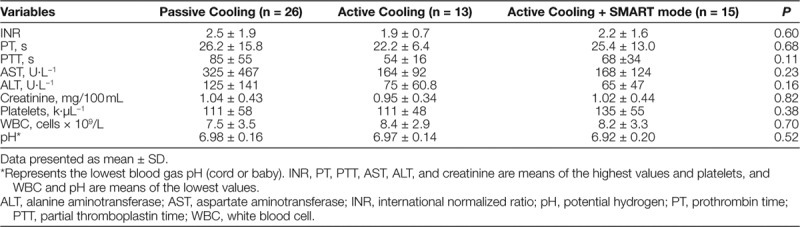
Laboratory Values During Cooling and Warming

**Table 4.**
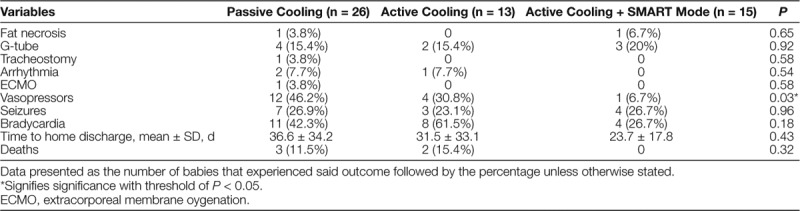
Health Outcomes of Babies by Protocol

## DISCUSSION

To provide infants the most neurodevelopmental protection with minimal side effects after perinatal HIE, medical teams must optimize the management of temperature targeting techniques during TH. This requirement can be particularly challenging in situations where TH is provided at a limited number of health care centers regionally, as is the case in our state. For transported patients, delays in initiation of treatment may lead to diminished therapeutic benefits, whereas overcooling can lead to increased mortality.^6,7,19^ To address this, multiple studies have evaluated the impact of active or passive TH during transport.^12,13,20–23^ Although evidence supports that active hypothermia during transport is safe, efficacy is likely impacted by the approach utilized.^24^

Studies have demonstrated that employing active hypothermia with a servo-controlled device leads to a larger percentage of patients hospitalized within the target temperature range, shorter times to stabilization of temperatures, fewer temperature readings outside of the target temperature range, and less overcooling when compared with passive hypothermia.^12,21^ Alternatively, those that compared passive to active hypothermia maintained with ice or gel packs found there to be more infants admitted within therapeutic range; however, these results were more modest compared with the studies evaluating active cooling with servo-controlled devices.

Additionally, when ice or gel packs were used for active TH, overcooling was a prevalent problem and, in some cases, occurred more frequently than with passive TH.^13,20,22,23^ Our finding that active TH on transport was associated with the more rapid achievement of target temperatures is not unexpected given the results of prior studies. It supports our continued use of servo-controlled hypothermia for transport.

Although using servo-controlled cooling blankets provides an automated mechanism for managing the core temperatures for infants during TH, some devices and modes function in differing ways, which can affect treatment.^15,16,25^ Due to this variability, considerations should be given to the modality used during TH and its effect on the quality of the delivery of therapy. Data from our study support the change from Auto mode to Gradient 10 with SMART mode in our population. This mode provided more stable temperatures within the goal therapeutic range and an overall decrease in the difference between core and peripheral temperatures. This change is associated with a lower incidence of side effects of treatment, including subcutaneous fat necrosis.^11,14^ Unfortunately, our QI evaluation was limited by the inability to detect significant improvements in the adverse health outcomes of HIE due to our small sample size. However, in a retrospective analysis, Filippi et al^14^ found a reduction in subcutaneous fat necrosis when patients were treated with a mode that gradually adjusts the temperature gradient when compared with patients treated with Auto mode.

The combined measures of changing to active cooling with a servo-controlled device on transport and utilizing Gradient 10 with SMART mode decreased the incidence of temperatures out of therapeutic range by half. This change is a noteworthy improvement considering the more recent randomized clinical trial completed by Shankaran et al^19^ in 2017, which demonstrated increased mortality when TH is provided for longer periods at colder temperatures. Although survivors had fewer disabilities, the increased risk of mortality supports strict adherence to the current recommended therapeutic goal temperature of 33.5°C.^19^

One unexpected observation of the study was a decrease in vasopressor support with each protocol change, which may comment on the better regulation of temperatures as a result of the implementations. Decreased cardiac output and bradycardia are expected side effects of TH, which can be exacerbated by overcooling. Alternatively, the observed differences in vasopressor use were the result of changes in hypotension management and not the improvements in temperature regulation. However, there were no ongoing systematic changes in practice to hypotension management in our NICU that overlapped with the study period. Notably, our sample size is relatively small, and the noted changes in vasopressor use could be incidental findings and not relative to clinical care.

Overall, the findings from this single-site QI study have the potential to inform other facilities when they are creating or modifying their protocols for TH. It is important to note that this information is only applicable to sites that are using high-tech servo-controlled whole-body cooling devices. Despite the recommendation that TH only be provided at experienced health care facilities with ample resources, there is a notable shift to providing this treatment at less-experienced sites with limited resources.^11^ Our data suggest that resources and experience are necessary to maximize benefits and minimize harm. It may be in the patient’s best interest to be transferred to a site with these resources and experience.^12,13^ Further, the gradual and refined treatment using the Gradient and SMART modes, which in this QI study led to more stable thermal control, would be very difficult, if not impossible, to achieve with lower-tech methods.

## CONCLUDING SUMMARY

Our data support the hypothesis that changing from passive to active TH on transport and optimizing cooling device settings led to the earlier achievement of target temperatures and more temperature stability throughout TH. These practice changes were associated with less frequent vasopressor use during TH. Similar strategies to optimize temperature control in patients undergoing TH for HIE may be relevant to other facilities providing TH.

## DISCLOSURE

The authors have no financial interest to declare in relation to the content of this article.

## ACKNOWLEDGMENTS

The authors wish to thank The University of Maryland School of Medicine and the Program for Research Initiated by Students and Mentors (PRISM) for their support and contribution to this project. The devices used in this study were utilized in a manner consistent with Food and Drug Administration clearance.
